# Twelve Tips for Re-imagining Problem-based Learning in Medical Education for the COVID-19 Era and Beyond

**DOI:** 10.15694/mep.2021.000110.1

**Published:** 2021-05-05

**Authors:** Hoi Yan Corliss Wong, Julie Yun Chen, Kendrick Co Shih

**Affiliations:** 1 Li Ka Shing Faculty of Medicine; 2 Department of Family Medicine and Primary Care; 3 Department of Ophthalmology

**Keywords:** Problem-based learning, COVID-19, e-learning, digital literacy, technology

## Abstract

This article was migrated. The article was marked as recommended.

In 2020, the COVID-19 pandemic led to an almost overnight adoption of online learning across medical schools worldwide. The initial experience proved jarring and challenging for students and medical educators alike, with neither side familiar with the new tools thrusted upon them. Over time, it became apparent that the adaptations made catalysed a long overdue systemic re-examination of the way medical education has been, and should be, delivered. So far, COVID-19 has served as a strong impetus for education institutes to incorporate commercially available and widely accessible communication platforms, including Zoom, to effectively deliver didactic lectures and interactive tutorials in the digital space. At our institution, the University of Hong Kong Li Ka Shing Faculty of Medicine (HKUMed), one such example is the problem-based learning (PBL) tutorial, the format of which has largely remained unchanged since its introduction two decades ago. By closely examining our offline-to-online transition, we have been able to identify certain pitfalls when attempting to translate traditional tutorials to the digital space; key considerations concerning the online facilitator-learner experience, and enhancements for more effective learning. In this article, we present twelve tips rooted in our experience as learners and teachers that should make PBL sessions more fruitful for everyone involved.

## Introduction

The start of the global COVID-19 pandemic necessitated the introduction of physical distancing measures and the cessation of face-to-face learning at education institutions worldwide. According to the United Nations’ policy brief on ‘Education during COVID-19 and beyond’, pandemic-related school closures have impacted nearly 94% of the world’s student population ( United Nations, 2020). Faced with mounting uncertainties over a timeline for the pandemic to be controlled, there was a near overnight global shift from face-to-face lessons to online learning.

Located in one of the first cities outside of Mainland China to be impacted by the COVID-19 pandemic, the University of Hong Kong Li Ka Shing Faculty of Medicine (HKUMed) was one of the first adopters of widespread online learning in medical education. Our early efforts in adapting clinical skills teaching, bedside learning, and professional development have been documented in the literature ( Choi, Ho and Smith, 2020; Shih *et al.*, 2020a; Tsang *et al.*, 2020; Tsang, Shih and Chen, 2021). We also began to recognise that certain skills, such as direct ophthalmoscopy, could not be taught effectively through this online format ( Shih *et al.*, 2020b). We then turned our attention to problem-based learning (PBL), a core feature of our curriculum. Since its adoption by our faculty in 1998, the principles and format of conducting paper cases have remained largely the same. By using popular videoconferencing platform Zoom (California, USA), our initial plan was to attempt to replicate the offline format online, however, it quickly became apparent that this approach would not suffice. Over time, we observed how individual facilitators were adapting their teaching practices to overcome various barriers, and institutional changes were quick to follow. Now, over a year since the current pandemic reportedly began, the dust has finally begun to settle, enabling educators and institutions to start thinking more deeply about re-conceptualising previous models of learning and their modus operandi. The goal has arguably moved on from retaining a sense of old normalcy to creating a new system that is far better than what preceded the pandemic.

In our experience, PBL tutorials, which have traditionally been face-to-face and socially constructivist in nature ( Gewurtz *et al.*, 2016), required some of the most substantial adjustments to our practice. By blending our experience with international phenomena and relevant literature, we have devised 12 tips to improve upon the traditional PBL format and explore productive engagement with disruption to achieve a ‘new better’. We sincerely hope that fellow medical educators and faculties will find these recommendations practicable and be inspired to think about PBL in a new light.

## Tip 1: Establish institutional digital protocol

Concepts such as digital citizenship ( Mossberger, Tolbert and McNeal, 2007) and network etiquette ( Shea and Shea, 1994) imply the presence of social codes of conduct that dictate conventional and respectful practices in the online arena. As more diverse and complex technological functions begin to be utilised in higher education, these norms may require continuous revisions to reflect such changes. For instance, the potential to ‘Zoom-bomb’ by entering a meeting without permission ( O’Flaherty, 2020) or record a meeting without the host’s knowledge through screen recording software like Camtasia (Techsmith, USA), means it is time to reconsider what teaching staff should expect of online learners and vice versa. A digital protocol that clearly outlines the expected online behaviour of students can help to reinforce existing professional standards and also introduce aspects pertaining solely to digital education. While explicit rules and recommendations are no guarantee of professional behaviour, they may clarify misconceptions and set explicit standards for this highly digitised age.

Our faculty’s “E-learning Rules and Etiquette” incorporates the following elements, and may provide a helpful framework for those seeking to establish institutional digital protocols of their own ( HKUMed, 2020):

1. Punctuality and general time management
2. Positive attitude to learning
3. Appropriate attire during online learning
4. Availability of the necessary technology and equipment
5. Protecting intellectual property rights on the webspace
6. Standardised use of full names for display and a requirement to keep webcams turned on during didactic lectures and interactive tutorials

The presence of an e-learning protocol emphasises the need to treat the virtual classroom with the same respect as face-to-face teaching. The overall aim is a positive learning environment with a focus on collaborative and mutually respectful discussion. In practice, however, this can be difficult to reinforce given the more casual setting of the home environment and perceived distance between the student and the teacher.

## Tip 2: Conduct recursive cycles of needs assessments and action

Within education, needs assessments are conducted to reveal gaps between current practices and desired practices ( Corbett and Redding, 2017), such that solutions may be derived to bridge the gap between the two. In particular, the shift to online learning has highlighted the inadequacies of previous needs assessments, or in some cases the absence of such assessments, especially in identifying individuals who require technological support. When new technologies are introduced to educational settings, it is essential to consider both learners’ and teachers’ respective levels of digital literacy, self-efficacy, and accessibility. These user characteristics may be revealed by various types of needs assessments, including surveys, self-assessments, and gap-analyses ( Grant, Chambers and Jackson, 1999).

Once specific needs have been identified, interventions can be carried out to address them appropriately ( Barr, Glennerster and Le Grand, 1989). Further needs assessments following the intervention will then assist in directing future targets of action, thereby kickstarting repetitive cycles of assessment and intervention. Understandably, the extent to which this can be conducted is subject to time, resources, and staffing at individual faculties. Nevertheless, by striving to act upon the most prevalent and pertinent issues ( Altschuld and Witkin, 1999), educational activities can become increasingly aligned with the abilities and capacities of those involved.

One such example encountered within our faculty was the previous lack of high-speed Internet access and audio-visual equipment within the teaching hospital compounds, which housed the academic offices of a number of our clinical teaching staff. An initial in-house survey carried out in mid-2020 identified the need to expand the Internet bandwidth to facilitate e-learning. A follow-up survey revealed the need to upgrade or add audio-visual equipment to facilitate better online teaching experiences. These findings prompted an increased involvement of technical staff to ensure higher quality Internet access and smoother operations during teaching.

## Tip 3: Provide technology education for PBL facilitators

A sizeable proportion of facilitators can be described as ‘digital immigrants’, having grown up in times when digital devices and the internet were far less ubiquitous ( Prensky, 2001). However, not all ‘digital immigrants’ are created equal, as facilitators can vary in their technology self-efficacy, actual abilities, and eventual technology use. Hence, it may be worth implementing baseline digital competencies to ensure they are able to execute their technology-based responsibilities effectively. In preparation for our faculty-wide online PBL implementation, we hosted a webinar on effective online PBL facilitation which covered the following topics:

1. Basic PBL pedagogical principles
2. Practical tips in terms of computer set-up and being mindful of ‘Zoom fatigue’
3. Recommendations for conducting online PBL effectively e.g. sharing screen, annotating, polling, break-out rooms
4. Tips to enrich online PBL teaching with additional web-based tools, including Mentimeter (Mentimeter, Sweden), Kahoot! (Kahoot!, Norway), Google Docs (Google, USA)
5. Sharing from early adopters of online PBL on challenges and how to overcome them

The webinar was supplemented by additional small group training workshops, in which experienced facilitators led the group to role-play a PBL case, followed by debriefing. Alternatively, we have found distributing user guides or instructional videos to be more flexible, self-directed methods for achieving the same aim.

## Tip 4: Incorporate pedagogical technologies in a deliberate manner

The current state of technology use within medical education has been described as one of over-utilisation ( Khong *et al*., 2020). At this rate, continued reliance on online technology for much, if not all, of medical teaching appears unsustainable. Given this overshoot, it is all the more vital to ensure that any new technology incorporated is not only purposeful, but able to enrich student learning. Most importantly, it should lie reasonably within the boundaries of facilitators’ expected capabilities. Institutions and individual facilitators should strive to be selective and resourceful in their approach to new technology to avoid using it for its own sake ( Borsheim, Merritt and Reed, 2008).

In addition, while studies have pointed towards the potential for enhanced knowledge and skill acquisition associated with digital PBL, there is currently limited evidence on other aspects, including negative effects, satisfaction, attitudes, and cost effectiveness ( Car *et al.*, 2019). Given these practical considerations play an integral role in planning as well as the overall PBL experience, they should be allocated appropriate thought and attention in both research and practice.

## Tip 5: Foster meaningful student-educator partnership

Nowadays, the rise of student-centred education means teachers increasingly view students as partners in the learning process. As such, students are no longer restricted to the role of passive consumers but rather active participants to be involved in decision-making processes ( Harrington, Flint and Healey, 2014). At HKUMed, we have been hosting regular online PBL fora, where students and staff (case writers, directors, facilitators) are given the opportunity to engage in bi-directional exchange and feedback. Along the same lines, we have also introduced a “Take a Moment..to Talk to a Teacher” series, in which individual staff members are allocated timeslots to converse with smaller groups of students in a mixed virtual-physical format.

A more recent addition to our faculty is the ‘med.co’ social platform, launched in 2021, to serve as a game changer in communication between teachers and students. The platform is powered by Workplace (Facebook, USA), a closed version of Facebook (Facebook, USA), providing a safe place for social interactions, community building and collaborative work within institutions. Central to the new way of communication is the availability of a companion app for direct messaging, which by facilitating more open and constructive discussion may change the educator-student partnership for the better. We have chosen to include all PBL groups and their respective facilitators on the platform.

## Tip 6: Update the PBL case database to reflect students’ modern realities

As technologies and ways of living continue to change at an accelerating rate ( Roser and Ritchie, 2013), case writers should ideally incorporate relevant modifications into their case content, with the aim of creating more relatable scenarios anchored in modern reality. The reasoning behind this tip lies in the philosophy of ‘authentic learning’ ( Herrington, 2014) in which learning is rooted in “real-world tasks, content and context” ( Roach, Tilley and Mitchell, 2018).

Several features to incorporate are suggested below:

• Scenarios relating to recent events
• Novel scientific findings, technologies, and corresponding changes in clinical practice
• The modern patient, including newer mannerisms and behaviours
• Details of locations familiar to the students e.g. local training hospital

In authentic PBL cases, students are given more opportunities to draw from their own personal experiences and observations which may facilitate more meaningful discussion. While it is not necessarily feasible to produce new cases for every cohort, especially in real-time, existing paper cases should be periodically revised such that students are able to relate more, and potentially engage more as a result.

## Tip 7: Keep an eye on the time

The term ‘Zoom fatigue’ describes the feelings of tiredness, burnout and anxiety associated with overusing virtual communicative platforms ( Wolf, 2020). Multiple aetiologies for this unique phenomenon have been proposed, among them negative interpersonal perceptions due to audio delays ( Roberts and Francis, 2013; Johnson *et al.*, 2016) and diminished attentional capacity from virtual multitasking ( Lee, 2020). Besides, it is well established that prolonged screen time can leave users vulnerable to developing computer vision syndrome, with symptoms ranging from eyestrain and headaches to neck and shoulder pain (American Optometric Association, n.d.).

To prevent or minimise the above during online PBL, facilitators may consider the following solutions:

1. Schedule regular short breaks to allow themselves and their students to rest.
2. Encourage off-screen activities, such as drawing concept maps and flow diagrams pertaining to the case content.
3. Keep sessions within the allocated time frame.
4. Consider shorter tutorials compared to their face-to-face equivalents.

## Tip 8: Encourage higher-order answers from students

The advent and continued development of search engines has made it significantly easier for students to find large quantities of information. The speed at which search results are retrieved may promote surface learning as opposed to the type of critical, deep learning that PBL is supposed to imbue. If the goal of modern education is to increase learners’ capacities for problem solving and critical thinking ( Olszewski-Kubilius and Thomson, 2015; Elder and Paul, 2020), facilitators should aim to plant strategic seeds of cognitive dissonance ( Festinger and Carlsmith, 1959) and propose more integrative questions pertaining to the top tiers of Bloom’s Taxonomy ( Bloom, 1956) to stimulate students’ higher cognitive faculties. Beyond merely remembering and understanding, students should be motivated to analyse and accommodate potentially conflicting pieces of information to derive an original answer ( Anderson and Bloom, 2001).

Critical engagement can be facilitated by giving students a chance to first discuss the question raised by the PBL facilitator amongst themselves in sub-groups of 2 or 3, in the form of Zoom breakout rooms, before returning to the main room. This can be done in their own format or through a think-pair-share (TPS) collaborative learning strategy approach ( Lyman, 1981):

1. Think - Each student individually considers the response to the question.
2. Pair - Each student is paired with another one or two students to discuss their thoughts with each other.
3. Share - Students return to share and consolidate their findings with the whole PBL group.

## Tip 9: Take the opportunity to cultivate telemedicine skills

The 2020 US State of Telemedicine Report revealed that utilisation of telehealth services is on the rise, with the proportion of American physicians recognising ‘telemedicine’ as a skill increasing by almost 40% in the past year alone ( Doximity, 2020). Globally, the telemedicine market is expected to increase in value by 130 billion US dollars before 2026 ( Stewart, 2020). Given these upward trends, it would be wise to begin incorporating telemedicine training into medical curricula if yet to do so.

Early on in the pandemic, our faculty identified the need for our students to be trained in telemedicine practices ( Tsang *et al.*, 2020; Tsang *et al.*, 2021). What began as simple adjustments to transfer the bedside clinical teaching experience to the webspace turned into a fully-fledged faculty initiative in training core telemedicine competencies.

To us, the online PBL setting appears to be an ideal environment to ease students into telemedicine and associated skills of virtual communication and webside manner. Through role-play and carefully constructed scenarios embedded within PBL cases, students can start to learn the telemedicine’s fundamentals to navigate healthcare environments of increasing complexity ( Bamidis, Angelidis and Kaldoudi, 2006). While PBL is likely to return to face-to-face when circumstances allow, faculties should consider reserving a number of PBL sessions for online telemedicine education.

## Tip 10: Continue to experiment and explore well after the pandemic

This pandemic has been a period of immense experimentation and innovation, with many medical educators trialling new approaches they perhaps never would have attempted otherwise. For instance, in an effort to reduce detachment and disengagement, Morawo, Sun and Lowden (2020) began incorporating individual-based anonymous quizzes and non-competitive audience polling sessions into online residency training. They found these methods to be effective in stimulating active learning and recommended for their continued use in live virtual learning environments beyond COVID-19. Likewise, HKUMed facilitators have been experimenting with similar technologies in the context of PBL.

As batches of vaccinations continue to be distributed, the end of the pandemic looks within reach. Having said this, educators must not forget the advances made during this period, nor should the receptive approach to unfamiliar pedagogical technologies many adopted be abandoned once a state of ‘normalcy’ returns. With a more open mindset comes less resistance to exploring non-traditional forms of PBL, which in turn may pave new paths for the implementation of alternative approaches and further research. In a way, recent research investigating the use of escape rooms ( Pearcy, Guise and Heller, 2019) and virtual reality ( Abdullah, Mohd-Isa and Samsudin, 2019) in PBL tells us the future is already here.

## Tip 11: If in doubt, keep it simple

Amidst all this talk about technology and innovation, it can be easy to neglect the value of simpler, less technology-based methods. To give an example, a study comparing high- and low-fidelity simulated Advanced Life Support training concluded that the former merely led to overconfidence among its participants ( Massoth *et al.*, 2019). Moreover, the learning curve associated with using new technologies may waste time and paradoxically detract students from the actual content to be learned ( Dontre, 2020), not to mention the accessibility issues that students with connection issues and/or less technologically advanced devices may face ( Johnson *et al.*, 2016). Therefore, faculties should take special care to acknowledge the downsides of technology before implementing en masse, and if ever in doubt, to keep it simple.

## Tip 12: Prepare for further disruption by researching widely

Medical educators must expect disruption and be proactive in forecasting future needs instead of solely reacting to them ( Burk, 2008). This means using disruption as an impetus to seek out the latest literature, best practices, and trends to anticipate needs well in advance ( Pilcher, 2016). Alongside more conventional sources of information used in medical education research, unconventional ones should be considered as a way to uncover new findings and perspectives (see Table 1).

**Table 1.** Conventional and unconventional sources for medical educators

Conventional	Unconventional
Academic literature e.g. journals, books, reports Formal conferences e.g. AMEE, ASME Inter-institutional exchange	Social media e.g. Reddit, Twitter, LinkedIn Video and podcast platforms e.g. YouTube, Spotify Online platform publishers e.g. Medium.com

It is worth noting the quality of unconventional sources are often not as standardised due to lower barriers for information dissemination. Even so, the absence of traditional academic standards should not result in their immediate dismissal as potentially valuable references ( Radia and Stapleton, 2009). Moreover, fewer barriers to quick publication and dissemination can mean that the information is up to date and reflective of the most recent observations and practices in the field. That being said, as with all sources, educator-researchers should remain vigilant in their critical appraisal, especially when seeking evidence to reshape, reform and revise current systems. In this way, conventional and unconventional resources may be used simultaneously and complementarily, without limiting oneself to the confines of non-disrupted tradition.

## Conclusion

The COVID-19 pandemic made 2020 a whirlwind year for medical institutions worldwide. At the same time, it has culminated in fresh new perspectives on how to move forward in a progressively digitised age. For our faculty, it became increasingly evident that our approach to PBL required modifications to better reflect the future that our students will eventually be living and practising in. By weathering each wave of disruption, we have faith that new pedagogical frameworks for PBL will emerge stronger and more aligned with students’ needs. Forget returning to normal; it is time we create a ‘new better’.

## Take Home Messages

• PBL is set within an institution’s education ecosystem, meaning changes made to PBL alone are unlikely to succeed unless the proper foundations have been laid.
• Maximise the chances of successful implementation of technology by careful evaluation, conducting needs assessments, and encouraging student-educator communication.
• Productively and proactively engage with disruption, treating it as a motivator of change.

## Notes On Contributors

**Hoi Yan Corliss Wong** is a 4 ^th^ year medical student at the Li Ka Shing Faculty of Medicine, the University of Hong Hong. She earned a BSc in Medical Education from the Queen Mary University of London. She currently serves as Director of Pedagogy and Research at Med Ed Laboratory HK, a student-centred virtual laboratory specialising in artificial intelligence systems and peer-assisted learning. ORCID: https://orcid.org/0000-0001-7619-7821.

**Julie Yun Chen** is Assistant Dean of Learner Wellbeing and a Clinical Associate Professor in Family Medicine and Primary Care at the Li Ka Shing Faculty of Medicine, the University of Hong Kong. She is also programme director of the faculty’s Bau Institute of Medical and Health Sciences Education. ORCID: https://orcid.org/0000-0002-7444-6182.

**Kendrick Co Shih** is Director of Student Affairs and a Clinical Assistant Professor in Ophthalmology at the Li Ka Shing Faculty of Medicine, the University of Hong Kong. He is a faculty certified PBL educator and works to train and accredit new academic staff. ORCID: https://orcid.org/0000-0001-6255-2941.
