# Supplementary material for: Twelve Tips for Re-imagining Problem-based Learning in Medical Education for the COVID-19 Era and Beyond
Source: MedEdPublish (2016). 2021 May 5;10:110. [Version 1] doi: 10.15694/mep.2021.000110.1 (PMC10939515; doi:10.15694/mep.2021.000110.1)
Supplement: Peer review history

# Reviewer report: Reviewer response for version 1

**DOI:** 10.21956/mep.19103.r27542

**Published:** 2021-10-03

**Authors:** Balakrishnan Nair

**Affiliations:** 1 Centre for Medical Professional Development and University of Newcastle

This review has been migrated. The reviewer awarded 4 stars out of 5 PBL is transactional learning to create transformational learning. As the authors state , the covid pandemic has affected small group, traditional PBL, learning. However the pandemic also taught us to be problem solvers and innovators. So this article about transforming PBL in IT platforms and the tips to do this , is very helpful for digital immigrants. The tips to reduce zoom fatigue is applicable for all meetings . I certainly learned from this article and I am sure others will do tooAs the authors state, we were force to disrupt , but even in the post pandemic era, we can adapt some of these innovations .

Reviewer Expertise:

NA

No decision status is available

# Reviewer report: Reviewer response for version 1

**DOI:** 10.21956/mep.19103.r27541

**Published:** 2021-06-18

**Authors:** Barbara Jennings

**Affiliations:** 1 Norwich Medical School

This review has been migrated. The reviewer awarded 4 stars out of 5 I found this to be a very engaging and well-written article, with the benefit of a thorough bibliography. The tips and cited literature will be of interest to any teachers and curriculum planners who use case-based learning, and PBL in particular. I really appreciated one of the introductory statements:“The goal has arguably moved on from retaining a sense of old normalcy to creating a new system that is far better than what preceded the pandemic.”Beginning with a discussion of protocols and etiquette (Tip 1) is important. After decades of application, while there may be a lot of subtle heterogeneity in the classroom, I think there is a shared understanding of the key features of a collegiate face-to-face PBL tutorial. We do not have a shared understanding yet of what digital PBL means however, so discussion and exploration is necessary as we consider explicit models. Taking account of unequal access for learners to technology/ and a suitable home environment for study may be one consideration that could be expanded on in a revised version for tip 11. The faculty training guidance offered in Tip 3 is very helpful though, with the suggested use of synchronous webinar training supplemented with guides for asynchronous further study and elaboration.Tip 6 about updating case-vignettes to reflect real world scenarios (and to perhaps to promote cultural competence? as well as advances in medical science) is sage advice, as is the guidance in tip 8 to capitalise on video-conference tools to encourage higher order discussion. The pandemic may have prompted all of us to refresh our curricula in a useful way.It is true that “Medical educators must expect disruption and be proactive in forecasting future needs instead of solely reacting to them” and the necessary expansion the size of healthcare professional training cohorts (across the globe) will be an ongoing disruptor that will necessitate nimble action from well-trained clinical educators.

Reviewer Expertise:

NA

No decision status is available
